# Cortisol Dose-Dependently Impairs Migration and Tube-like Formation in a Trophoblast Cell Line and Modulates Inflammatory and Angiogenic Genes

**DOI:** 10.3390/biomedicines9080980

**Published:** 2021-08-09

**Authors:** Talita Guerreiro Rodrigues Húngaro, Marcos F. Gregnani, Thaís Alves-Silva, Florian Herse, Natalia Alenina, Michael Bader, Ronaldo C. Araújo

**Affiliations:** 1 Nephrology Program, Laboratory of Genetics and Exercise Metabolism, Biophysics Department, Federal University of São Paulo (UNIFESP), São Paulo 04039-032, Brazil; talita.guero@gmail.com; 2 Molecular Biology Program, Laboratory of Genetics and Exercise Metabolism, Biophysics Department, Federal University of São Paulo (UNIFESP), São Paulo 04039-032, Brazil; mgregnani@hotmail.com (M.F.G.); thais.silva13@unifesp.br (T.A.-S.); 3 Max-Delbrück Center for Molecular Medicine (MDC), Robert-Rössle-Str. 10, 13125 Berlin, Germany; florian.herse@charite.de (F.H.); alenina@mdc-berlin.de (N.A.); 4 Experimental and Clinical Research Center (ECRC), a Cooperation of Charité—Universitätsmedizin Berlin and Max Delbrück Center for Molecular Medicine (MDC), Lindenberger Weg 80, 13125 Berlin, Germany; 5 Berlin Institute of Health, 10178 Berlin, Germany; 6 German Center for Cardiovascular Research (DZHK), Partner Site Berlin, 10117 Berlin, Germany; 7 Max Delbrück Center of Molecular Medicine, Charité University Medicine, Charitéplatz 1, 10117 Berlin, Germany; 8 Institute for Biology, University of Lübeck, Ratzeburger Allee 160, 23562 Lübeck, Germany

**Keywords:** cortisol, extravillous trophoblast, angiogenesis, cell migration, kinin receptors

## Abstract

Several stimuli can change maternal hormone levels during pregnancy. These changes may affect trophoblastic cells and modulate the development of the embryo and the placental tissue itself. Changes in cortisol levels are associated with impaired trophoblast implantation and function, in addition to other pregnancy complications. This study aims to analyze the effects of low and high doses of cortisol on an extravillous trophoblast cell line, and the effects of various exposures to this hormone. SGHPL-4 cells were treated with cortisol at five doses (0–1000 nM) and two exposures (continuous: 24 h/day; and intermittent: 2 h/day). In intermittent treatment, cortisol acted mainly as an anti-inflammatory hormone, repressing gene expression of kinin B1 receptors, interleukin-6, and interleukin-1β. Continuous treatment modulated inflammatory and angiogenic pathways, significantly repressing angiogenic factors and their receptors. Cortisol affected cell migration and tube-like structures formation. In conclusion, both continuous and intermittent exposure to cortisol repressed the expression of inflammatory genes, while only continuous exposure repressed the expression of angiogenic genes, suggesting that a sustained increase in the levels of this hormone is more harmful than a high short-term increase. Cortisol also impaired tube-like structures formation, and kinin receptors may be involved in this response.

## 1. Introduction

The placenta, a highly differentiated maternal–fetal organ intended exclusively for offspring’s development, is a linking tissue between the mother and the fetus. Besides being essential for embryo nutrition and development, it also acts as a barrier, preventing high levels of hormones or other peptides from reaching the fetal circulation [1,2]. During placental development and in a sequence of complex events, cytotrophoblast cells differentiate into multinucleated syncytiotrophoblasts and also aggregate to form anchoring villi [3], which will later give rise to extravillous trophoblasts (EVTs), with an invasive phenotype [4,5].

One of the main characteristics of EVTs is the ability to migrate into the maternal spiral arteries and invade the venous and lymphatic vessels [6]. EVTs are responsible for anchoring the placenta in the uterus and for the invasion of uterine vessels [7]. Changes in their functions can lead to serious pregnancy complications, such as preeclampsia, which presents shallow trophoblastic invasion and failure in vascular transformation [8,9,10].

The SGHPL-4 cell line represents EVTs, developed from the human placenta in the first trimester of pregnancy [11,12] and is widely used in invasion and migration studies [13,14,15,16,17]. Additionally, the SGHPL-4 cell line has characteristics of normal EVTs and the invasive capacity similar to primary EVTs [13] and has therefore been previously recommended as an EVT model for in vitro studies [18].

In addition to the essential role of trophoblasts, several adaptations are required for a pregnancy to be successful, and many of these physiological adjustments are driven by the mother’s hormones [1]. Alterations in hormone or other peptides levels in the mother’s blood can affect trophoblastic cells and modulate the development of both the embryo and the placental tissue itself. Cortisol, the main glucocorticoid (GC), is one of the key hormones for the maintenance and evolution of pregnancy [2] and for preparing the fetus to the extrauterine life [19,20]. It is a hormone secreted in a pulsatile manner that follows a circadian rhythm and is sensitive to environmental factors, such as light and darkness periods, sleep, exercise, food deprivation, and diseases [21,22,23]. At adequate levels, this hormone is essential for healthy gestational development, including the stimulation of human chorionic gonadotropin (hCG) secretion, suppression of uterine natural killer cells [24], and promotion of human leucocyte antigen-G (HLA-G) expression in trophoblasts [25].

Despite their important functions, exposure to synthetic glucocorticoids or physiological stress during pregnancy has been negatively related to fetal development and pregnancy complications [1,2,26,27,28,29]. Changes in cortisol levels have been associated with impaired trophoblast implantation and function [30,31], including migration, proliferation and invasion of EVTs [32], inhibition of cytokine-prostaglandin signaling, induction of apoptosis [24], restriction of intrauterine growth [26,33], low birth weight, and preeclampsia [1,34].

Considering the importance of maintaining adequate hormone levels during pregnancy, there are mechanisms that protect the fetus from excess maternal GCs, such as the enzymatic activity of 11-hydroxysteroid dehydrogenase type 2 (HSD11B2), which is crucial for the metabolism of cortisol, transforming it into an inactive form [1,33,35]. When this mechanism is not sufficient, cortisol levels can reach supraphysiological values over a long period, thus promoting the aforementioned disorders [2,34]. The HSD11B2 enzyme is found in placental cells. Initially, it is limited to syncytiotrophoblasts, and at around 10–12 weeks of pregnancy, this enzyme is also found in cytotrophoblasts and EVTs [36].

It is important to note that each trimester of human pregnancy has a specific inflammatory profile: in the first trimester, cytokines such as interleukin-1β (IL1B) and interleukin-6 (IL6) are necessary for the implantation process and for proper EVT function [37,38], and the lower levels of cortisol contribute to this, since it is a hormone with anti-inflammatory activities [26]. On the other hand, in the third trimester, baseline cortisol increases, contributing to prevent pulmonary collapse through surfactant production and also contributing to the induction of childbirth [39]. It is also worth noting that placental cells, including EVTs [40], express a high level of glucocorticoid receptor (GR) [41], which is capable of responding to changes in cortisol levels.

In human pregnancy, appropriate trophoblast implantation and proliferation is a determining factor for the fetal and maternal outcome. Taking into account the actions promoted by cortisol at both physiological and supra-physiological levels, as well as the essential function of trophoblasts for gestational development, especially EVTs, little is known about the effect of cortisol on these cells, as well as the effects of this hormone on early pregnancy [24]. Thus, this study aimed to analyze the effects of treatment with low and high doses of cortisol on SGHPL-4 cells, as well as the effects of continuous and intermittent exposure to this hormone on gene expression and cellular function.

## 2. Materials and Methods

### 2.1. Cell Culture and Cortisol Treatment

The first trimester EVT cell line, SGHPL-4, a kind gift from Judith E. Cartwright (St. George’s University of London, London, UK), was used. Based on its phenotypic and functional characteristics, this cell line has been recommended as a cell model to study EVT function in vitro [18]. The cells were cultivated in HAM’s F10 Medium (Biochrom GmbH FG0715 with 1.2 g/L NaHCO_3_, 10 mg/L Phenol red, stable glutamine, and low endotoxin) and supplemented with 10% of heat-inactivated fetal bovine serum (FBS) and 1% of penicillin-streptomycin (Thermo Fisher, Waltham, MA, USA) at 37 °C with 5% CO_2_ and 21% O_2_. All experiments were conducted considering optimal passage range for SGHPL-4 (between cell passages 18 to 25) [18]. 

The cells were plated at a density of 1.2 × 10^5^ cells/well in 6-well flat-bottom plates. The next day, the medium was replaced by a starvation medium, with 0.5% FBS and 1% antibiotics. The cortisol (CORT; Sigma-Aldrich, St. Louis, MO, USA) used for treatments was a ready-to-use solution prepared in 100% methanol (1 mg/mL). After 24 h starvation, the cells were treated with 100, 250, 500, or 1000 nM cortisol in the culture medium. In the control group, the volume of methanol (vehicle) used was the same volume of cortisol applied in the highest treatment dose, and the control group appears in the result graphs as “0 nM”. The doses chosen for treatment were equivalent to physiological cortisol levels at baseline (100 and 250 nM), stress (500 nM), and supra-physiological dose (1000 nM) [24].

**Figure 1:**
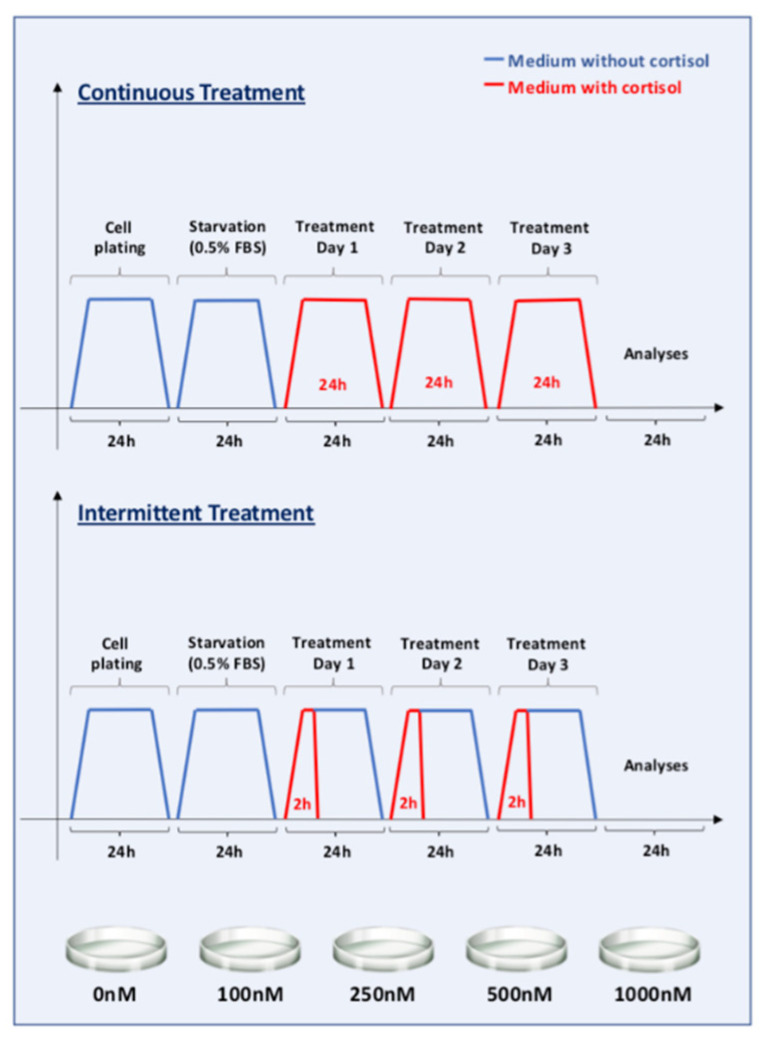
Study design. The first trimester extravillous trophoblast cell line SGHPL-4 was divided into 2 main groups: “continuous” and “intermittent” exposure to cortisol. Each of these groups was divided into another 5 groups according to the cortisol dose (0, 100, 250, 500, or 1000 nM). The cells were plated on the first day, and the next day, the medium was replaced with one with only 0.5% FBS (fasting). After 24 h starvation, the cells were treated with cortisol (a ready-to-use solution prepared in 100% methanol) in the culture medium, and the control cells were supplemented with the same volume of methanol (vehicle). Continuous exposure groups received the cortisol doses 24 h/day, for 3 days, and intermittent exposure groups received cortisol in the culture medium for 2 h/day, also for 3 days. The culture medium was changed daily.

The cells were divided into 10 groups, 5 groups of “continuous” exposure, and 5 groups of “intermittent” exposure to cortisol. Continuous exposure was performed in order to mimic a chronic increase in cortisol levels, while intermittent exposure simulated conditions that increase the level of this hormone in a pulsatile/intermittent manner, such as physical exercise or several other acute situations. Continuous exposure groups received the cortisol doses 24 h/day, for 3 days, and intermittent exposure groups received cortisol in the culture medium for 2 h/day, also for 3 days (Figure 1). Control groups were treated only with the vehicle, without cortisol. The culture medium was changed daily in all groups, in order to ensure the same handling process and also due to cortisol half-life. After treatment, the cells were collected and stored at −80 °C until RNA extraction.

### 2.2. RNA Extraction and RT-qPCR

RNA was extracted with Trizol (#15596026, Thermo, Waltham, MA, USA) according to the manufacturer’s instructions after processing the SGHPL-4 cells in a homogenizer (FastPrep^®^-24, MP Biomedicals LLC, Irvine, CA, USA). The RNA quality and integrity were assessed by electrophoresis and observation of the 260/280nm absorbance ratio detected by nucleic acid spectrophotometer (NanoDrop 1000, Thermo Scientific, Waltham, MA, USA). Complementary DNA (cDNA) was synthesized using cDNA Reverse Transcription kit (#4368814, Thermo, Waltham, MA, USA) using random primers and following the manufacturer’s instructions. The qPCR reaction was performed using 9ng of cDNA and Hot FirePol Evagreen qPCR Mix, ROX (Solis Biodyne, #08-24-00001), 10 µL reaction volume in QuantStudio5 equipment (Applied Biosystems). For primer designing, we used Primer3 (primer3.ut.ee; last accessed on 5 May 2021) and Primer Blast (ncbi.nlm.nih.gov/tools/primer-blast/; last accessed on 5 May 2021). Heterodimers, self-dimers, and hairpins were checked at https://www.idtdna.com/calc/analyzer/; last accessed on 5 May 2021. The values were normalized to reference gene 18S and calculated using the 2^−ΔΔCt^ method; results are expressed as fold change of the control value. For qPCR, all samples were analyzed in duplicate. The primers used in this study are shown in Table A1.

### 2.3. MTT—Cell Proliferation Assays

In order to measure the cell proliferation rate and cell viability, a colorimetric MTT assay was performed. For this experiment, 1 × 10^3^ cells were added to 100 µL of medium in each well of a 96-well microplate and cortisol treatments were carried out as described in item 2.1 of this manuscript. The doses chosen for this experiment were 0 (control), 250, 500, and 1000 nM cortisol in a continuous exposure (24 h/day, for 3 days). The next day, a MTT assay was performed as previously reported [42], with some adaptations, as described below.

For the MTT assay, 10 µL of the 5 mg/mL MTT reagent (Thiazolyl Blue Tetrazolium Bromide, Sigma-Aldrich, St. Louis, MO, USA) were added to each well of a 96-well microplate and incubated at 37 °C for 6 h. After this period, when the intracellular punctate purple dye was clearly visible under the microscope, 100 µL of dimethyl sulfoxide (DMSO) were added to all wells. After a 20 min incubation at room temperature, the absorbance was measured at 570 nm wavelength under an automatic microplate reader. In this assay, the optical density is proportional to the number of living cells.

### 2.4. Wound Healing Assay

The wound healing assay is a standard in vitro technique used to evaluate cell migration. Using a pipette tip, a cell-free area is created in a confluent monolayer of SGHPL-4 cells, in order to see whether the exposure to the cell-free area would induce these cells to migrate into the gap, as previously described [43]. The cells were injured using standard p200 pipette tips, and after removing the medium, the wells were washed once with PBS, and medium was replaced with one containing the respective cortisol treatment. This trophoblast cell line was treated with cortisol (250, 500, and 1000 nM) and compared to control (cortisol-free medium). The experiment was carried out three times. Photos and measurements were taken after 48 h of exposure. The cell-free areas at 0h and 48h time-points were quantified by Image J as pixels and expressed as wound healing closure (%) [43].

### 2.5. Tube-Like Formation Assay

Tube-like formation assay was performed with SGHPL-4 cells as previously described [44]. First, 10 µL of Matrigel (Corning™ Matrigel™-Corning, NY, USA) were pipetted into each well of a 15-well plate (µ-Slide Angiogenesis–Ibidi) and incubated for 30 min at 37 °C with 5% CO_2_.

**Figure A1:**
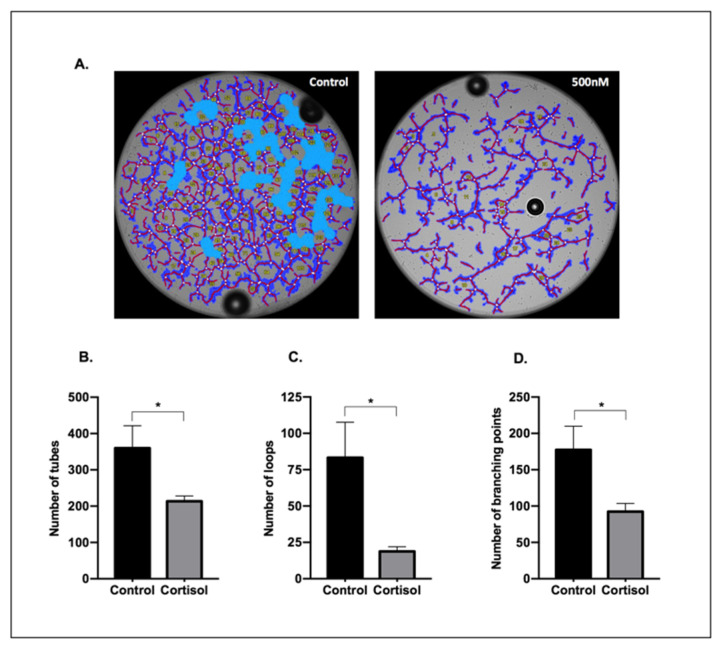
Tube-like structures formation—a pilot experiment. Representative images of tube formation (**A**) and the quantification of the number of tubes (**B**), loops (**C**), and branching points (**D**) of SGHPL-4 cells treated with vehicle (0 nM) or cortisol (500 nM) in the medium. Images were obtained after 5 h of stimulation. Data were compared with t-test and Shapiro–Wilk test; * *p* < 0.05. Data are presented as mean ± SEM. The quantification of tubes, loops, and branching points was done with the WimTube Software (Wimasis, Munich, Germany).

Then, the cells were plated (8 × 10^3^ cells/well diluted in 50 µL of medium with 2% of FBS and 1% of antibiotics) on coated wells in the presence or absence of cortisol (500 nM) and/or 1 µM of Des-Arg9-Bradykinin (DBK), a kinin B1 receptor agonist. After 5 h, the wells were observed under inverted phase contrast microscope (Zeiss, Jena, Germany). The number of branching points, tubes, and loops was quantified with the WimTube Software (Wimasis, Munich, Germany) considering the counts per image derived from independent experiments (*n* = 4) performed in triplicate. A pilot experiment was carried out to standardize the assay (Figure A1).

### 2.6. Statistical Analyses

GraphPad Prism 7 for Mac OS X, version 7.0a, 2 April 2016 was used for statistical analyses. Data are shown as mean ± standard error of the mean (SEM). Data were compared by one-way ANOVA or two-way ANOVA followed by Tukey multiple comparisons test. Statistical significance was considered for *p* < 0.05 (GraphPad Software, La Jolla, CA, USA, www.graphpad.com, accessed on 10 May 2021).

## 3. Results

### 3.1. Continuous Treatment Represses NR3C1 Gene without Modulations in HSD11B2

**Figure 2:**
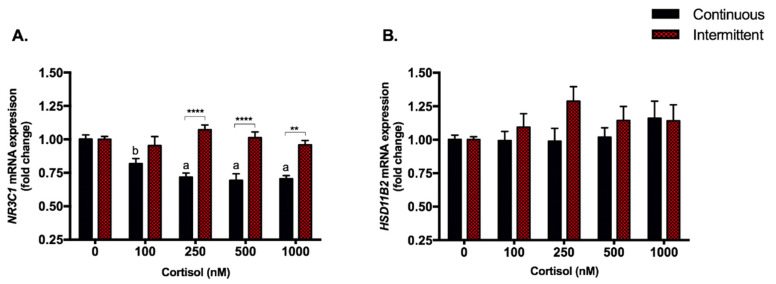
Glucocorticoid receptor and HSD11B2 enzyme gene expression. RT-qPCR of *NR3C1* (**A**) and *HSD11B2* (**B**) in SGHPL-4 cells treated with continuous or intermittent exposure to cortisol (100, 250, 500, 1000 nM) and vehicle (0 nM) in the medium. Data were compared by two-way ANOVA with Tukey multiple comparisons test. Interaction between (continuous or intermittent) exposure to cortisol and cortisol dose was found for *NR3C1* (*p* < 0.0001). Data are presented as mean ± SEM. ** *p* < 0.01; **** *p* < 0.0001; for continuous exposure: b, *p* < 0.05 and a, *p* < 0.0001 vs. control. *NR3C1*, Glucocorticoid receptor; *HSD11B2*, 11-hydroxysteroid dehydrogenase type 2. Cell treatment was repeated at least three times.

All four cortisol doses of continuous treatment were able to repress the expression of *NR3C1*, the glucocorticoid receptor gene for up to 30%: 100 nM: *p* < 0.05; 250, 500 and 1000 nM *p* < 0.0001 (Figure 2A). No modulation was observed with intermittent treatments (Figure 2A). The expression of the *HSD11B2* gene, which encodes the enzyme responsible for cortisol inactivation, was not modulated by any dose of both treatments (Figure 2B).

### 3.2. The Anti-Inflammatory Effect of Cortisol Was Found to Be Similar with Both Treatments; However, Kinin Receptors Responded Differently to Each Exposure

**Figure 3:**
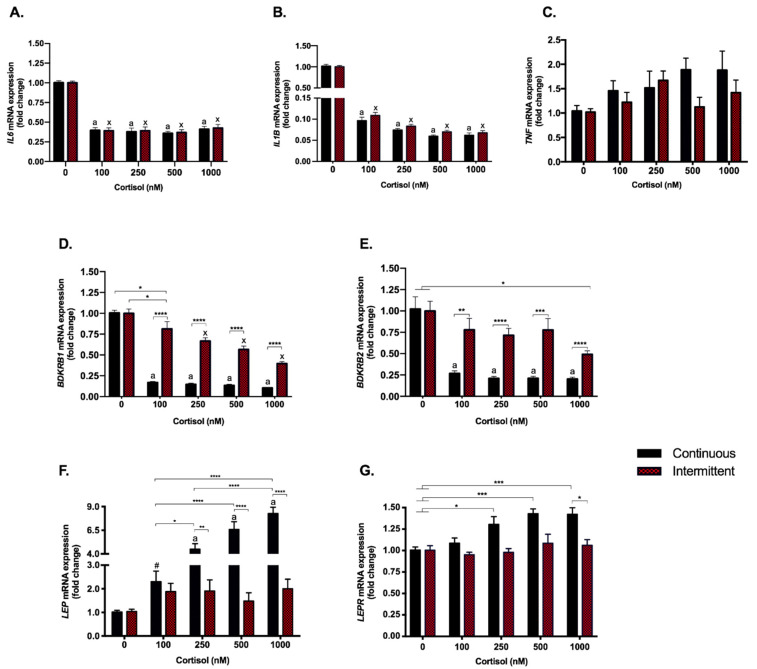
Inflammatory genes–gene expression. RT-qPCR of *IL6* (**A**), *IL1B* (**B**), *TNF* (**C**), *BDKRB1* (B1R) (**D**), *BDKRB2* (B2R) (**E**), *LEP* (**F**) and *LEPR* (**G**) in SGHPL-4 cells treated with continuous or intermittent exposure to cortisol (100, 250, 500, 1000 nM) and vehicle (0 nM) in the medium. Data were compared by two-way ANOVA with Tukey multiple comparisons test. Interaction between (continuous or intermittent) exposure to cortisol and cortisol dose was found for B1R (*p* < 0.0001), B2R (*p* = 0.0066), and *LEP* (*p* < 0.0001). Data are presented as mean ± SEM. * *p* < 0.05; ** *p* < 0.01; *** *p* < 0.001; **** *p* < 0.0001; #, *p* = 0.9356; for continuous exposure: a, *p* < 0.0001 vs. control; for intermittent exposure: x, *p* < 0.0001 vs. control. *IL6*, Interleukin 6; *IL1B*, Interleukin 1β; *TNF*, Tumor necrosis factor α; *B1R*, Kinin B1 receptor; *B2R*, Kinin B2 receptor; *LEP*, Leptin; *LEPR*, Leptin receptor. Cell treatment was repeated at least three times.

The inflammatory genes, *IL6* and *IL1B*, responded similarly to both treatments: the four doses of both continuous and intermittent treatments repressed their expression (*p* < 0.0001). The repression observed for *IL6* was ≃60% and even higher for *IL1B*, ≃90% (Figure 3A,B). Tumor necrosis factor α (*TNF)* was not modulated at all by both treatments (Figure 3C). The Kallikrein-Kinin System genes, i.e., *BDKRB1* and *BDKRB2*, which encode the kinin B1 (B1R) and B2 (B2R) receptors, were consistently repressed by continuous treatment (*p* < 0.0001 for all doses; B1R by 80–90% and B2R by ≃75%) (Figure 3D,E), while the repression by intermittent treatment was less pronounced, B1R by 13–61% and B2R by 22–51%, and dose-dependent for the B1R (Figure 3D,E).

### 3.3. Continuous Treatment Modulates Leptin (LEP) and Leptin Receptor (LEPR) Expression

After continuous treatment, the three highest cortisol doses were shown to increase the expression of *LEP* (250 nM, 500 nM and 1000 nM, *p* < 0.0001) in a dose-dependent manner from 4.5 up to 8.3 times (Figure 3F). Despite no statistical difference was observed in continuous exposure with 100 nM of cortisol (two-way ANOVA), *LEP* expression was 2.3 times higher compared to control. The expression of *LEPR*, which encodes the leptin receptor (OB-R) was increased for 250, 500 and 1000 nM doses (≃40%) of continuous treatment, with no differences between them (Figure 3G). No modulation was observed for *LEP* or *LEPR* with intermittent treatment.

### 3.4. Angiogenesis Pathway Genes Are Modulated by Continuous Exposure to Cortisol Even at Low Doses. PlGF and KDR (VEGFR-2) Are Also Repressed by the Highest Dose in the Intermittent Exposure

**Figure 4:**
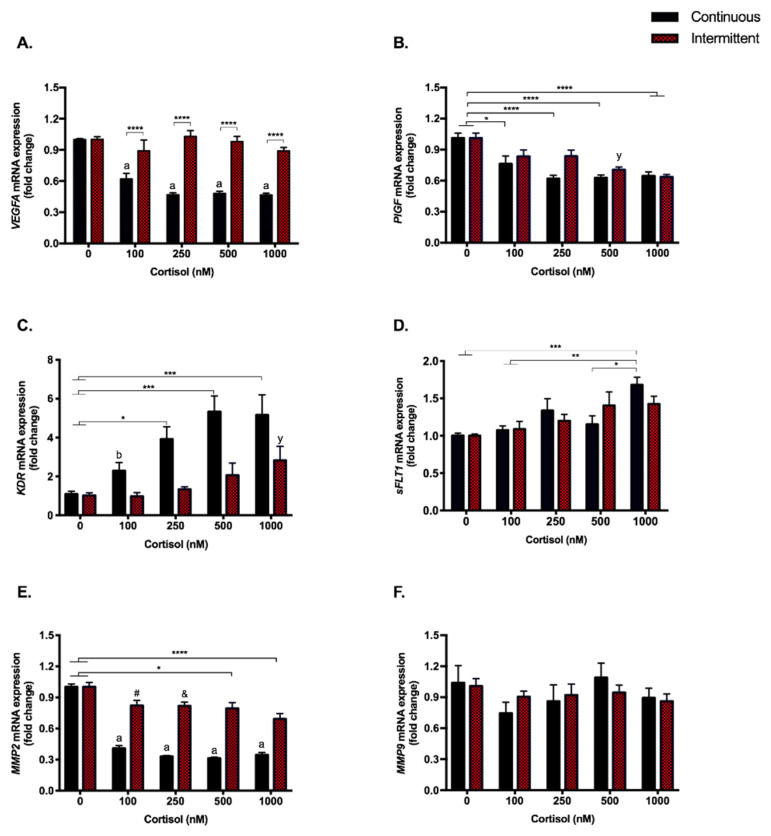
Angiogenic genes–gene expression. RT-qPCR of *VEGFA* (**A**), *PlGF* (**B**), *KDR* (VEGFR-2) (**C**), *sFLT1* (soluble VEGFR-1) (**D**), *MMP2* (**E**), and *MMP9* (**F**) in SGHPL-4 cells treated with continuous or intermittent exposure to cortisol (100, 250, 500, 1000 nM) and vehicle (0 nM) in the medium. Data were compared by two-way ANOVA with Tukey multiple comparisons test. Interaction between (continuous or intermittent) exposure to cortisol and cortisol dose was found for *VEGFA* (*p* < 0.0001), and *MMP2* (*p* < 0.0001). Data are presented as mean ± SEM. * *p* < 0.05; ** *p* < 0.01; *** *p* < 0.001; **** *p* < 0.0001; #, *p* = 0.0674; &, *p* = 0.0549; for continuous exposure: b, *p* < 0.05 and a, *p* < 0.0001 vs. control; for intermittent exposure: y, *p* < 0.05 vs. control. *VEGFA*, Vascular endothelial growth factor A; *PlGF*, Placental growth factor; *KDR* (VEGFR-2), Vascular endothelial growth factor receptor 2; *sFLT1* (soluble VEGFR-1), soluble Vascular endothelial growth factor receptor 1; *MMP2*, Matrix metalloproteinase-2; *MMP9*, Matrix metalloproteinase-9. Cell treatment was repeated at least three times.

Continuous exposure to cortisol strongly repressed (*p* < 0.0001) the expression of the angiogenesis-related genes for vascular endothelial growth factor A (*VEGFA*) (≃47%, Figure 4A), placental growth factor (*PlGF*) (24–37%, Figure 4B), and matrix metalloproteinase 2 (*MMP2*) (31–40%, Figure 4E), at almost all doses. On the other hand, intermittent treatment modulated the expression of some of the aforementioned genes only at the highest doses: *PlGF* (30–37%) and *MMP2* (21–31%) (500 nM, *p* < 0.05; 1000 nM, *p* < 0.0001).

**Figure A2:**
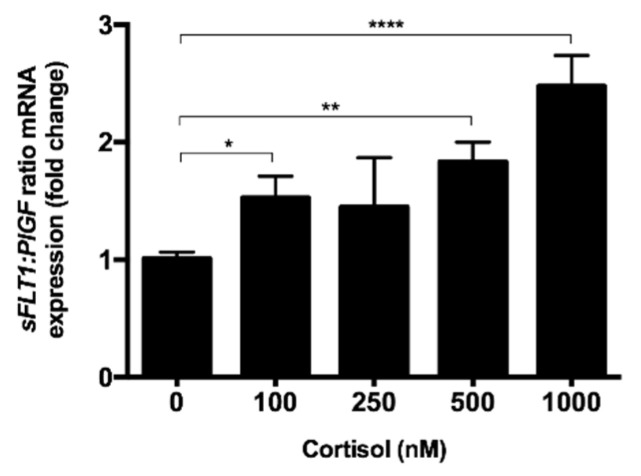
RT-qPCR of sFLT:PlGF ratio. *sFLT1*:*PlGF* mRNA ratio in SGHPL-4 cells treated with continuous exposure to cortisol (100, 250, 500, 1000 nM) and vehicle (0 nM) in the medium. Data were compared by Mann–Whitney test. Data are presented as mean ± SEM. * *p* < 0.05; ** *p* < 0.01; **** *p* < 0.0001. sFLT1 (soluble VEGFR-1), soluble Vascular endothelial growth factor receptor 1; PlGF, Placental growth factor. Cell treatment was repeated three times.

The receptor for these angiogenic factors, i.e., VEGFR-2 (*KDR*), which is capable of binding to VEGF and PlGF and is responsible for the pro-angiogenic signaling pathway, showed the opposite effect: all doses of continuous treatment increased the expression of this receptor up to five times, while there was no modulation by intermittent treatments (Figure 4C). Soluble VEGFR-1, encoded by an alternative transcript of the *FLT1* gene (*sFLT1*), an anti-angiogenic receptor that also binds to VEGF and PlGF and blocks the signaling, was increased (*p* < 0.001) only with continuous treatment at 1000 nM (≃70%; Figure 4D). The *sFLT1/PlGF* ratio was also increased in the continuous treatment (Figure A2). No modulation was observed for *MMP9* (Figure 4F).

**Figure A3:**
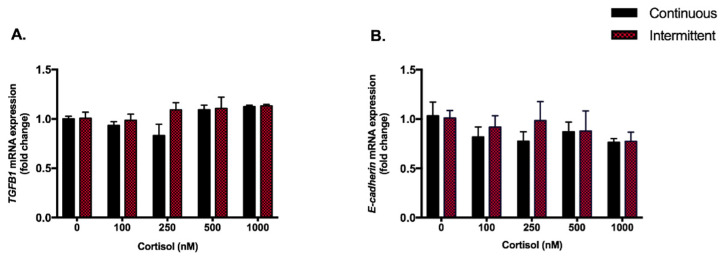
RT-qPCR. *TGFB1* (**A**) and *E-cadherin* (**B**) mRNA in SGHPL-4 cells treated with continuous or intermittent exposure to cortisol (100, 250, 500, 1000 nM), and vehicle (0 nM) in the medium. Data were compared by two-way ANOVA with Tukey multiple comparisons test. Data are presented as mean ± SEM. TGFB1, Transforming growth factor β1. Cell treatment was repeated three times.

Considering the modulations on MMP2, we also looked to other epithelial-to-mesenchymal transition (EMT) related genes, such as TGFβ1 and E-cadherin, but none of them were modulated by neither continuous treatment nor higher doses of intermittent treatment (Figure A3).

### 3.5. Cortisol Treatment Represses the Expression of Chemokine CXCL12 and Its Receptor

**Figure 5:**
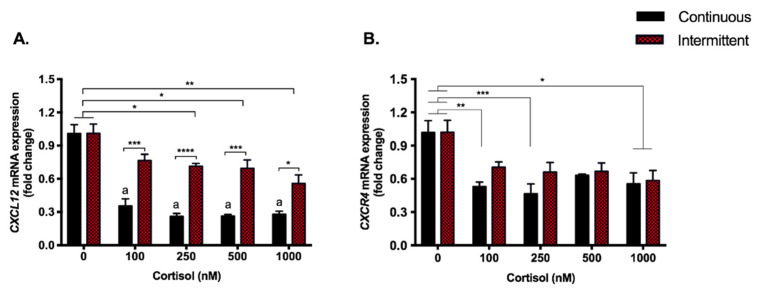
Chemokine gene expression. RT-qPCR of *CXCL12* (**A**) and *CXCR4* (**B**) in SGHPL-4 cells treated with continuous or intermittent exposure to cortisol (100, 250, 500, 1000 nM) and vehicle (0 nM) in the medium. Data were compared by two-way ANOVA with Tukey multiple comparisons test. Interaction between (continuous or intermittent) exposure to cortisol and cortisol dose was found for CXCL12 (*p* = 0.0027). Data are presented as mean ± SEM. * *p* < 0.05; ** *p* < 0.01; *** *p* < 0.001; **** *p* < 0.0001; for continuous exposure: a, *p* < 0.0001 vs. control. *CXCL12* (SDF-1), C-X-C motif chemokine ligand 12 (Stromal cell-derived factor 1); *CXCR4*, C-X-C chemokine receptor type 4. Cell treatment was repeated at least three times.

The C-X-C Motif Chemokine Ligand 12 (*CXCL12*) gene expression was repressed by both treatments, although this modulation was stronger with continuous exposure to cortisol, which repressed it by 65–74% (*p* < 0.0001) compared to 24–45% by the intermittent exposure (Figure 5A). Regarding C-X-C chemokine receptor type 4 (*CXCR4)*, all doses used in the continuous treatment repressed gene expression (37–54%; *p* < 0.0001), while only the highest dose had this effect in the intermittent treatment (42%; *p* < 0.05) (Figure 5B).

### 3.6. Cortisol Had No Impact on the Viability of the SGHPL-4 Cell Line

**Figure 6:**
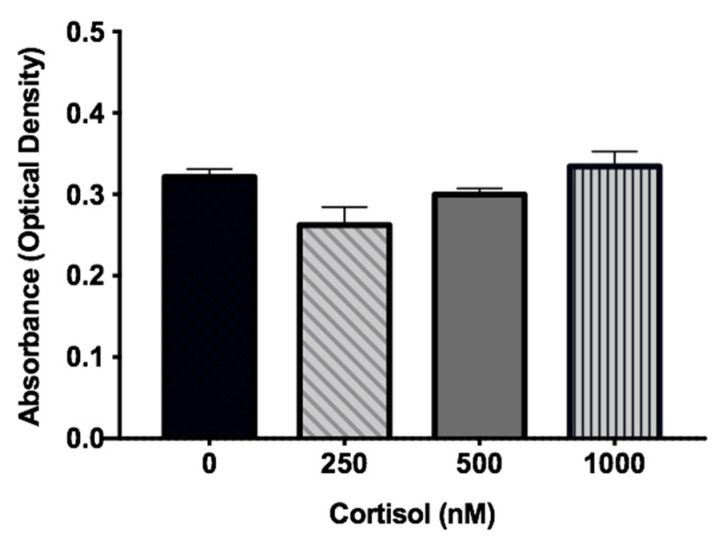
MTT assay. Cell viability assay in SGHPL-4 cells treated with the three highest doses (250, 500, and 1000 nM) of cortisol in continuous exposition. Data were compared by one-way ANOVA with Tukey multiple comparisons test. Data are presented as mean ± SEM.

After analyzing the gene expression results, we speculated whether the effects promoted by continuous cortisol treatment were directly related to the time and dose of the hormone or if they could be due to cell viability, since cortisol could be interfering with cell proliferation and survival. Therefore, the MTT assay was performed, which confirmed that cell viability was not impacted by any treatment (Figure 6).

### 3.7. Cortisol Affects Cell Migration in a Dose-Dependent Manner

**Figure 7:**
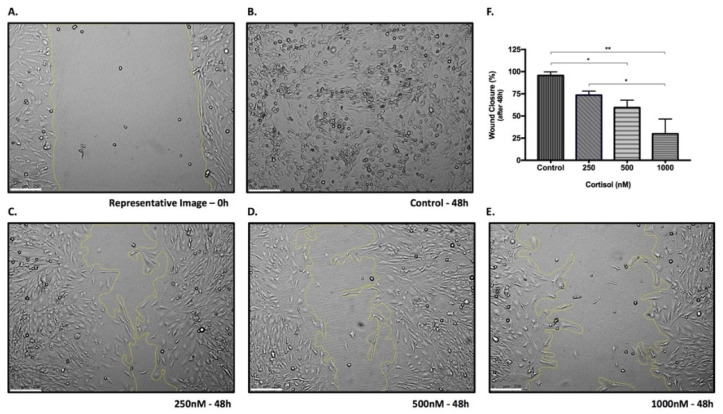
Wound healing assay. Representative image of 0 h time-point (**A**) and representative images after 48 h of SGHPL-4 cells stimulation: vehicle (control) (**B**), 250 nM (**C**), 500 nM (**D**) and 1000 nM (**E**) of cortisol. Quantification of wound closure (%) after 48 h of stimulation (**F**). Data were compared by one-way ANOVA with Tukey multiple comparisons test. Data are presented as mean ± SEM. * *p* < 0.05, ** *p* < 0.01. Cell treatment was repeated three times. Scale bar = 250 µm.

EVTs are invasive cells that migrate to remodel the vessels in the maternal–fetal interface. This characteristic depends on the degradation of the extracellular matrix and also on the action of angiogenic growth factors, genes that have been repressed by cortisol treatment. In order to investigate whether the protein functions were also impaired, the wound healing assay was performed with the three highest doses of cortisol used in continuous treatment, and cortisol was found to affect SGHPL-4 migration in a dose-dependent manner. Figure 7 shows that the cortisol dose was directly related to the wound healing area at the end of the experiment: the higher the dose of the hormone, the larger the area that was not occupied by the cells.

### 3.8. Cortisol Treatment Reduces the Formation of Tube-Like Structures and DBK Modulates This Response

**Figure 8:**
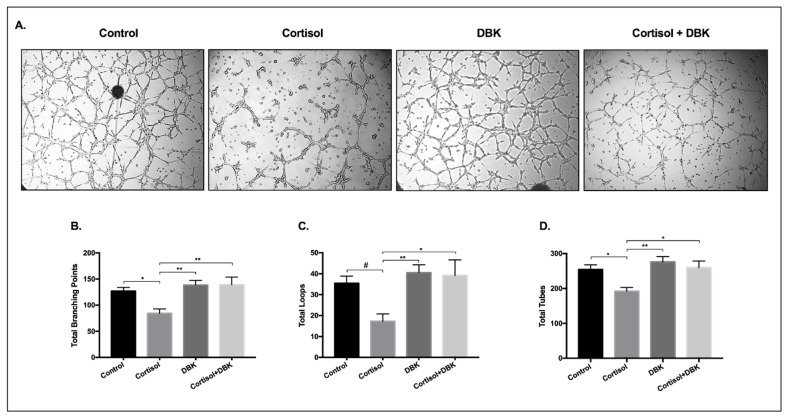
Tube-like structures formation. Representative pictures of tube-like structures (**A**) and the quantification of total branching points (**B**) total loops (**C**), and total tubes (**D**) after a 5-h treatment of SGHPL-4 cells with vehicle (0 nM), cortisol (500 nM), and/or DBK (1 µM) in the medium. The quantification of tubes, loops, and branching points was performed using the WimTube Software (Wimasis, Munich, Germany). Data were compared by one-way ANOVA with Tukey multiple comparisons test; * *p* < 0.05, ** *p* < 0.01, # *p* = 0.0521. Data are presented as mean ± SEM. DBK, Des-Arg9-Bradykinin. Cell treatment was repeated at least three times.

The tube-like structures formation assay is often used as an in vitro angiogenesis measure to investigate the cells’ ability to form a network. This assay was performed on matrigel in the presence of 500 nM cortisol, which is considered a physiological stress dose. As shown in Figure 8B,D, the total number of branching points, as well as the number of tubes, were significantly reduced after cortisol treatment compared to the control (reduced by 33% and 24%, respectively; *p* < 0.05). In addition, a trend towards decrease in the number of loops was observed (*p* = 0.0521; Figure 8C).

Since kinin receptors have been previously related to angiogenesis [45,46] and also considering the response of these receptors to cortisol, the cells were treated with cortisol and DBK, a B1R agonist. We hypothesized that DBK would improve tube formation, but that was not observed. However, when the cells were treated with cortisol + DBK, the B1R agonist reversed the effect promoted by cortisol (Figure 8A).

## 4. Discussion

The present study showed that both intermittent and continuous treatments with cortisol are able to affect the SGHPL-4 trophoblast cell line. The effects of cortisol on the placental cells have been previously investigated, but there are important peculiarities to consider, such as cell lines (HTR8/svneo, Sw.71, BeWo, JEG3), synthetic or physiological hormone (dexamethasone, cortisol), dose used for the treatment, and last but not least, the objective of each investigation. Our study’s design consisted of treating SGHPL-4 EVTs with various cortisol doses within its physiological concentration range (100–600 nmol/L) [24] in order to analyze the differences between the same doses in different exposure patterns.

In our intermittent treatment, cortisol acted mainly as an anti-inflammatory hormone, repressing characteristic inflammatory genes, such as *IL6*, *IL1B* and *BDKRB1*. Continuous cortisol exposition, even at baseline and physiological stress doses, was capable of modulating both inflammatory and angiogenic pathways, suggesting that the exposure duration can be more relevant than the cortisol dose.

The effects of cortisol occur primarily through its binding to the glucocorticoid receptor (GR), a ligand-activated transcription factor. After that, cortisol can modulate gene transcription mainly through the interaction with the glucocorticoid response element (GRE) or other transcription factors, increasing or decreasing the expression of target genes [47]. In our cell treatments, continuous exposition to cortisol repressed *NR3C1* for up to 30%, while intermittent treatment did not affect the GR gene expression. This is expected since chronic stress or inflammatory conditions requiring long-term administration of GC can lead to repressed transcription of the *NR3C1* and to resistance to glucocorticoids [48].

The hormonal levels need to be rigorously balanced during pregnancy, including cortisol, which is essential for life, but can cause serious health issues when imbalanced (1, 35). The HSD11B2 enzyme is the main player in cortisol metabolism, this enzyme converts the bioactive cortisol into cortisone, which has much lower bioactivity and is considered the inactive form of cortisol [49]. In both continuous and intermittent treatments, *HSD11B2* was not modulated by any dose, not allowing us any inference regarding how it is being affected. Given that HSD11B2 is responsible for cortisol to cortisone conversion, the concentrations of these two steroids in the supernatant would be necessary to verify whether the activity of this enzyme has been modulated by both continuous and intermittent treatments in EVTs or not.

Beginning with genes from inflammatory pathways, *IL6* and *IL1B* responded similarly to both treatments and had their expression strongly repressed. On the other hand, *TNF* was not modulated in all groups from continuous and intermittent treatments, although it was possible to observe a trend towards an increase in the highest doses of continuous exposition. *TNF* as well as *IL6* and *IL1B* are glucocorticoid-sensitive genes that classically present a decreased transcription after cortisol exposition [50]. Since these three cytokines are induced by nuclear factor kappa B, which can also be repressed by cortisol [48], we were expecting a similar response profile, but it was not confirmed. Although we cannot explain this effect, divergence in the cytokine response has been previously reported by authors that showed modulation of IL6 and/or IL1B after stressor stimuli without modulation of TNF in in vivo [51] and in vitro studies [52].

The inflammatory milieu is necessary for embryo implantation. *IL6* together with *IL1B* and *BDKRB1*, were the inflammatory genes most consistently repressed, and all three are involved in important processes for pregnancy, such as modulation of protease functions and improvement of blastocyst implantation; therefore, changes in their levels are sufficient to impair pregnancy [53]. 

B1R is a receptor from the Kallikrein-Kinin System (KKS), a system widely known for its role in inflammatory processes [54,55] that has also been described as participating in angiogenesis [46,56]. The actions of the KKS are mediated through signaling by kinin B1 and B2 receptors, while B2R is constitutively expressed, B1R is inducible by inflammation, however, constitutively expressed in the placenta [46,57]. In our study, the *BDKRB1* and *BDKRB2* genes, which encode the B1R and B2R, respectively, were consistently repressed by continuous treatment, but also by intermittent treatment, albeit less pronounced. Additionally, the repression observed for B1R after intermittent exposition to cortisol was dose dependent, which did not happen for other inflammatory genes. Besides the role of kinins in inflammation, B1R and B2R signaling can induce VEGF and VEGFR-2 expression [56,58], and B2R also contribute to the transactivation of VEGFR-2 through phosphorylation [59]. B1R as well as B2R and VEGFR-2 signaling increase intracellular concentrations of Ca^2+^ and activate endothelial NO synthase, inducing cell migration and proliferation. Kinins have been shown to play a role in neovascularization in ischemic stress and tumor models, however, KKS knockout animals did not seem to have physiological vascular impairments. Despite the importance of the KKS in the process of new vessels formation, it is possible that the KKS does not play a crucial role in embryogenesis but modulates adaptations to ischemic stress [60], and may also be involved in the trophoblastic response to cortisol.

Lastly, in the inflammatory context, no modulation was found in *LEP* and *LEPR* after the intermittent treatment, however, while almost all genes were repressed after continuous exposure to cortisol, *LEP* expression increased in a dose-dependent manner from 2.3 up to 8.3 times, and an increase around 40% was observed in *LEPR* expression. This increase in leptin was also reported by Tzschoppe et al., 2011, who treated primary human cytotrophoblasts with 10 µM dexamethasone for up to 72 h, and found that the glucocorticoid incubation stimulates leptin production [61], which is also produced by placenta during pregnancy along with adipose tissue [61,62]. Leptin is a hormone related to the nutritional and pro-inflammatory state, but also involved in angiogenesis, healthy pregnancy, and early human development. Basak and Duttaroy, 2012, showed that leptin induces tube formation in first trimester EVTs, and also showed that this action was independent of VEGF [63], one of the main angiogenic factors. Additionally, leptin promotes trophoblast cell proliferation and survival [62]. Considering that, it is possible that the increase we found in the expression of *LEP* and *LEPR* genes may be a response to cortisol due to the repression we observed in genes related to angiogenic pathways to maintain tube formation.

Regarding angiogenic factors, *VEGFA* and *PlGF* were significantly repressed by continuous exposure to cortisol, but the expression of their receptors, *KDR* and *sFLT1*, was increased. While VEGFR-2 (*KDR*) is known to be the main receptor for the VEGF and PlGF angiogenic signaling pathways, the soluble receptor VEGFR-1 (*sFLT1*) also binds to these factors, but unlike VEGFR-2, it blocks signaling, and is therefore considered an anti-angiogenic receptor [64]. The increased expression of both receptors may be an attempt to compensate for the repressive effects promoted by cortisol on their ligands. Considering intermittent treatment, even at supra-physiological doses, it did not modulate angiogenic pathways, and for some of the evaluated genes (*KDR*, *PlGF* and *MMP-2*), only the highest dose resulted in repression of mRNA expression.

Interestingly, our data are in line with the changes found in some pregnancy complications, such as intrauterine growth restriction (IUGR) and preeclampsia. The levels of sFLT1 in maternal serum are increased in preeclampsia and the sFLT1:PlGF ratio is elevated in pregnant women before the clinical onset of preeclampsia, which is a predictive value for this pregnancy complication [65]. In our study, the continuous treatment with cortisol increased the *sFLT1*:*PlGF* mRNA ratio in almost all doses. Our data are also consistent with a previous publication that showed an increased *sFLT1*:*PlGF* mRNA ratio with vitamin D depletion in a rodent model for preeclampsia [66]. Likewise, leptin protein and mRNA levels are also increased in the placenta of women with preeclampsia [67].

For a successful vascular uterine remodeling, matrix metalloproteinases (MMPs) are also essential. Increases in MMP2 and MMP9 have been implicated in placentation and physiological adaptations in a healthy pregnancy, and the expression and/or activity of these MMPs may be altered during pregnancy complications, such as the decrease in MMP2 and MMP9 observed in hypertensive pregnancy and preeclampsia [68]. In our study, we did not find modulation in *MMP9*, but *MMP2* was repressed by both continuous (31–40%) and the highest doses of intermittent (21–31%) treatments, besides a trend towards decrease with the 100 nM and 250 nM doses. In early pregnancy, a low concentration of placental MMPs may affect the spiral artery remodeling, impacting the maternal-fetal perfusion [68]. Despite the relevance of MMP9, authors have shown that MMP2 could be more relevant in trophoblast invasion at the initial stage of early pregnancy [69].

The inflammatory milieu is necessary for cell motility and MMP induction. Cortisol has been shown to inhibit TGFβ1-induced EMT by others [70]. TGFβ1 inhibits trophoblastic migration and invasiveness by increasing inhibitors of MMP2, a marker for EMT, and by modulating E-cadherin [71]. The downregulation or repression of E-cadherin is linked to the migratory phenotype of trophoblast cells, including EVTs [72]. Given the above, and considering the repression found for *MMP2*, we hypothesized that cortisol could contribute to an increase in *TGFB1* and *E-cadherin* expression but neither continuous treatment nor the higher doses of intermittent treatment modulated the expression of both mRNAs, suggesting that the observed effects could be due to the modulatory effects of cortisol directly on inflammatory and angiogenic markers.

Since cortisol affects inflammatory and angiogenic genes essential for embryo implantation, we analyzed the chemokine CXCL12, also known as stromal cell-derived factor (SDF)-1. This is a homeostatic chemokine that binds mainly to CXCR4, but also to CXCR7 receptors, to induce intracellular signaling essential for processes such as placentation, implantation, and vascular remodeling in early pregnancy [73,74,75], in addition to inducing trophoblast invasion and placental angiogenesis [73,76]. CXCL12-mediated signaling in placental trophoblast cells occurs via many signaling pathways, such as ERK1/2, AKT, p38, JAK/STAT, PI3K/AKT/FOSL1, inducing vascular growth factors and leading to the utero-placental vascular remodeling. Placental trophoblasts, including EVTs, are the main source of CXCL12 [73]. Regarding the effects of cortisol on this chemokine in placental tissue or trophoblast cells, we have found no references in the literature, but other steroid hormones, such as estradiol and progesterone, are known to decrease the expression of CXCL12 [73]. This is in line with our findings since both continuous and intermittent treatments with cortisol repressed *CXCL12* expression, which is more accentuated with the continuous treatment (65–74% vs. 24–45%). The continuous exposition also modulated the expression of *CXCR4* (37–54%). It is possible that continuous exposure to cortisol represses signaling through this chemokine, which could be one of the pathways to reduce the growth factors essential for trophoblastic function.

EVTs are invasive cells that migrate to remodel the vessels in the maternal–fetal interface. This characteristic depends on the degradation of the extracellular matrix and also on the action of angiogenic growth factors, genes that have been repressed by cortisol treatment. In order to investigate whether the protein functions were also impaired, a migration assay was performed, and cortisol was found to affect SGHPL-4 migration in a dose-dependent manner. In addition, the difference between the effects of intermittent and continuous exposure to cortisol was not related to cell viability, since treatments with cortisol did not result in cell death, as assessed by the MTT assay.

Considering the cortisol effects on EVTs in the tube-like structures formation assay, a physiological stress dose of cortisol promoted a reduction in the number of tubes, loops, and branching points after 5h of exposure. According to previous publications, EVTs invade spiral arteries and alter their phenotype, acquiring endothelial-cell like capacities, thus promoting a network of tube-like structures [36,44,70]. Taking into account that kinins are peptides widely known for their role in the inflammatory process [54,55] and previously linked to angiogenesis and trophoblast function [46,56], we hypothesized that the B1R agonist, DBK, could increase tube-formation in SGHPL-4 cells. As shown in our results, this effect was not observed; however, when administered together with cortisol, DBK reversed the negative effects promoted by the hormone, suggesting that B1R could be involved in the anti-angiogenic response to cortisol. It is important to note that the tube formation assay needs to be carefully adjusted for each cell type, and SGHPL-4 cells show the best results within around 5 to 8 h of experiment, with almost all cells killed within 24 h.

Despite these relevant findings, it is important to highlight that our experiments with SGHPL-4 as first-trimester cell line were conducted at 21% O_2_, and the physiological oxygen concentration in early pregnancy is lower, 1% [77,78,79]. The high levels of oxygen can provoke oxidative stress resulting in cytokine alterations and endothelial dysfunction [4,80]. Furthermore, oxygen is a regulator of trophoblast proliferation [80]. Under physiological oxygen levels, cell function may be impacted differently by cortisol than when cultured in hyperoxic conditions.

Considering the differences in gene modulation by distinct exposures to cortisol, Reddy et al. [81] characterized genome-wide GR binding by combining GR binding and gene expression data applying a range of doses (100 pM–1 mM) and times of exposure (5 min to 4 h) to dexamethasone (DEX), a synthetic GC used to treat inflammatory diseases. Using a lung epithelial carcinoma cell line, the authors found that up-regulated genes were much more likely to have proximal GR binding than repressed genes (47% vs. 8%). They also found differences in the average time for response between up- and down-regulated genes. For the genes evaluated, 50% response occurred with 3 nM DEX, and they also found a faster response for up-regulation, although after 1h of exposure the percentage of total response was similar for both up-regulation and down-regulation. 

The results of these kinetic experiments are in line with the findings of other authors [82], who showed rapid gene induction and repression by synthetic cortisol. Given this, we believe that the different modulations observed with continuous and intermittent exposure to cortisol can be related to the repression or stimulation pathways of each target gene. Even considering that GR stimulation involves a series of regulatory actions and multifarious responses, the kinetic evaluation of differences in GR occupancy and angiogenic gene expression in trophoblast cells could improve the understanding of GC effects on these cells. As for future investigations, we would also like to emphasize the importance of evaluating the effects of different exposures to cortisol on epigenetic modifications in DNA [83,84], especially during the first trimester of pregnancy [85]. The study of these effects on gene regulation would strongly contribute to the current knowledge.

Given the above, we conclude that continuous exposure to cortisol represses inflammatory and angiogenic genes in a dose-dependent manner, which did not happen with intermittent treatment, suggesting that a sustained increase in this hormone is more harmful than a high short-term increase. In addition, cortisol treatment decreased cell migration and tube formation in SGHPL-4 cells, and kinin B1 receptors inhibit this response. Therefore, experiments evaluating the involvement of kinin receptors in the response promoted by cortisol in the context of angiogenesis and trophoblast implantation are promising and encouraged. Although the results presented in this study yields additional information on the response of EVTs to the main physiological glucocorticoid, the missing protein data for verification of the mRNA expression is a limitation of our work.

## Data Availability

Not applicable.

## Appendix A

**Table A1:** Base pair sequences of primers used in real-time PCR assays.

Gene	Primers
NR3C1	For: 5′-CGA CCA ATG TAA ACA CAT GCT-3 Rev: 5′-CCG TCC TTA GGA ACT GAA GAG-3′
HSD11B2	For: 5′-TCA AGA CAG AGT CAG TGA GAA ACG-3′ Rev: 5′-GGA ACT GCC CAT GCA AGT G-3′
18S	For: 5′-ACA CGT TCC ACC TCA TCC TC-3′ Rev: 5′-CTT TGC CAT CAC TGC CAT TA-3′
IL6	For: 5′-TGG CTG AAA AAG ATG GAT GCT-3′ Rev: 5′-TCT GCA CAG CTC TGG CTT GT-3′
IL1B	For: 5′-AGG GAC AGG ATA TGG AGC AAC AAG -3′ Rev: 5′-CAT CTT TCA ACA CGC AGG ACA GGT-3′
TNF	For: 5′-TGG CCC AGG CAG TCA GA-3′ Rev: 5′-GGT TTG CTA CAA CAT GG GCT ACA-3′
BDKRB1 (B1R)	For: 5′-CTG CAC AGA GTG CTG CCA ACA TT-3′ Rev: 5′-ACA CCA GAT CAG AGG CTG CCA GG-3′
BDKRB2 (B2R)	For: 5′-TCT GGC TTC TGG GCT CCG AG-3′ Rev: 5′-AGC GGC ATG GGC ACT TCA GT-3′
VEGFA	For: 5′-TACCTCCACCATGCCAAGTG -3′ Rev: 5′-GATGATTCTGCCCTCCTCCTT -3′
PlGF	For: 5′-TGC TGC GGC GAT GAG AAT C-3′ Rev: 5′-CCC TTG GGT CTC CTC CTT TC-3′
KDR	For: 5′-GCA TCT CAT CTG TTA CAG C-3′ Rev: 5′-CTT CAT CAA TCT TTA CCC C-3′
sFLT1	For: 5′-AAT CAG AGG TGA GCA CTG CAA C-3′ Rev: 5′-TGG TAC AAT CAT TCC TTG TGC TTT-3′
MMP2	For: 5′-CCC ACT GCG GTT TTC TCG AAT-3′ Rev: 5′-CAA AGG GGT ATC CAT CGC CAT-3′
MMP9	For: 5′-AGA CCT GGG CAG ATT CCA AAC-3′ Rev: 5′-CGG CAA GTC TTC CGA GTA GT-3′
LEP	For: 5′-CAT TGG GGA ACC CTG TGC GGA TTC-3′ Rev: 5′-TGG CAG CTC TTA GAG AAG GCC AGC-3′
LEPR	For: 5′-GTA AGA GGC TAG ATG GAC TGG GAT AT-3′ Rev: 5′-ATT CTC CAA AAT TCA GGT CCT CTC A-3′
CXCL12	For: 5′-ATT CTC AAC ACT CCA AAC TGT GC-3′ Rev: 5′-ACT TTA GCT TCG GGT CAA TGC-3′
CXCR4	For: 5′-GGG CAA TGG ATT GGT CAT CCT-3′ Rev: 5′-TGC AGC CTG TAC TTG TCC G-3′
TGFB1	For: 5′-ACC AAC TAT TGC TTC AGC TC-3′ Rev: 5′-TTA TGC TGG TTG TAC AGG G-3′
E-cadherin	For: 5′-GCC ACA TAC ACT CTC TTC TC-3′ Rev: 5′-CCA TCA CAG AGG TTC CTG-3′

NR3C1, Glucocorticoid receptor; HSD11B2, Hydroxysteroid 11-Beta Dehydrogenase 2; 18S,18S ribosomal RNA; IL6, Interleukin 6; IL1B, Interleukin 1β; TNF, Tumor necrosis factor α; B1R, Kinin B1 receptor; B2R, Kinin B2 receptor; VEGFA, Vascular endothelial growth factor A; PlGF, Placental growth factor; KDR (VEGFR-2), Vascular endothelial growth factor receptor 2; sFLT1 (soluble VEGFR-1), soluble Vascular endothelial growth factor receptor 1; MMP2, Matrix metalloproteinase-2; MMP9, Matrix metalloproteinase-9; LEP, Leptin; LEPR, Leptin receptor; CXCL12 (SDF-1), C-X-C motif chemokine ligand 12 (Stromal cell-derived factor 1); CXCR4, C-X-C chemokine receptor type 4; TGFB1, Transforming growth factor β1.
